# Cross-Sectional Study of 24-Hour Urinary Electrolyte Excretion and Associated Health Outcomes in a Convenience Sample of Australian Primary Schoolchildren: The Salt and Other Nutrients in Children (SONIC) Study Protocol

**DOI:** 10.2196/resprot.3994

**Published:** 2015-01-15

**Authors:** Carley A Grimes, Janet R Baxter, Karen J Campbell, Lynn J Riddell, Manuela Rigo, Djin Gie Liem, Russell S Keast, Feng J He, Caryl A Nowson

**Affiliations:** ^1^Centre for Physical Activity and Nutrition ResearchSchool of Exercise and Nutrition Research SciencesDeakin UniversityMelbourneAustralia; ^2^School of Exercise and Nutrition SciencesDeakin UniversityMelbourneAustralia; ^3^Centre for Environmental and Preventative MedicineWolfson Institute of Preventative MedicineQueen Mary University of LondonLondonUnited Kingdom

**Keywords:** sodium, dietary, sodium chloride, dietary, potassium, dietary, child, urine specimen collection, blood pressure, obesity, taste, Australia

## Abstract

**Background:**

Dietary sodium and potassium are involved in the pathogenesis of cardiovascular disease. Data exploring the cardiovascular outcomes associated with these electrolytes within Australian children is sparse. Furthermore, an objective measure of sodium and potassium intake within this group is lacking.

**Objective:**

The primary aim of the Salt and Other Nutrient Intakes in Children (“SONIC”) study was to measure sodium and potassium intakes in a sample of primary schoolchildren located in Victoria, Australia, using 24-hour urine collections. Secondary aims were to identify the dietary sources of sodium and potassium, examine the association between these electrolytes and cardiovascular risk factors, and assess children’s taste preferences and saltiness perception of manufactured foods.

**Methods:**

A cross-sectional study was conducted in a convenience sample of schoolchildren attending primary schools in Victoria, Australia. Participants completed one 24-hour urine collection, which was analyzed for sodium, potassium, and creatinine. Completeness of collections was assessed using collection time, total volume, and urinary creatinine. One 24-hour dietary recall was completed to assess dietary intake. Other data collected included blood pressure, body weight, height, waist and hip circumference. Children were also presented with high and low sodium variants of food products and asked to discriminate salt level and choose their preferred variant. Parents provided demographic information and information on use of discretionary salt. Descriptive statistics will be used to describe sodium and potassium intakes. Linear and logistic regression models with clustered robust standard errors will be used to assess the association between electrolyte intake and health outcomes (blood pressure and body mass index/BMI z-score and waist circumference) and to assess differences in taste preference and discrimination between high and low sodium foods, and correlations between preference, sodium intake, and covariates.

**Results:**

A total of 780 children across 43 schools participated. The results from this study are expected at the end of 2015.

**Conclusions:**

This study will provide the first objective measure of sodium and potassium intake in Australian schoolchildren and improve our understanding of the relationship of these electrolytes to cardiovascular risk factors. Furthermore, this study will provide insight into child taste preferences and explore related factors. Given the cardiovascular implications of consuming too much sodium and too little potassium, monitoring of these nutrients during childhood is an important public health initiative.

## Introduction

### Background

In most developed countries, salt (sodium chloride) is commonly added to the food supply [1]. Consequently, children frequently consume more dietary sodium than recommended [2], as sodium chloride is the main contributor to sodium intake (~90%) [3]. Consuming too much sodium during childhood is associated with raised blood pressure [4], which is a risk factor for future cardiovascular disease [5]. There is also some evidence to suggest that the combination of sodium and potassium in the diet is an important determinant of blood pressure levels during childhood [6]. For long-term gains in public health, it is important that dietary intakes of sodium and potassium are monitored in the pediatric population. This information can be used to guide future dietary interventions that aim to maintain healthy blood pressure levels across the lifespan and in turn reduce the burden of cardiovascular disease.

### Measuring Sodium and Potassium Intake

#### Overview

The use of 24-hour urinary electrolyte excretion is a reliable, objective method to estimate group intakes of sodium and potassium [7,8] and overcomes a number of limitations associated with self-reported dietary assessment methodologies.

#### Sodium Intake

Approximately 90-95% of ingested sodium is excreted in the urine [9]. Some sodium is lost through sweat; the amount is dependent on level of physical activity and exposure to heat [3]. Because of the high recovery of dietary sodium via urinary losses, 24-hour urine collection is considered the “gold-standard” method to determine sodium intake and its use is recommended in sodium assessment studies [10]. Due to the large intra-individual fluctuations in day-to-day sodium intake, it is necessary to complete at least seven 24-hour urine collections to assess an individual’s intake of sodium [11]. However, larger population studies generally utilize one 24-hour urine collection when attempting to describe the average sodium (salt) intake of groups [12] or to monitor changes in sodium intake in a study population [13].

Despite the robustness of 24-hour urine samples to objectively determine dietary sodium intake, it is difficult to get a representative number of people to complete due to the associated participant burden and logistical challenges. Alternatively, many studies, particularly large national nutrition surveys, assess sodium intake from 24-hour dietary recalls [14]. As with other nutrients, dietary assessment has a range of limitations including respondent bias and reliance on memory to recall food intake [15]. In the case of sodium, dietary recall assessment is further limited by the quality of food composition databases and the inability to quantify the amount of salt added during cooking and at the table [14]. For these reasons, dietary records tend to underestimate total sodium intake [16,17]. In developed countries, where the majority of salt comes from processed foods, the daily underestimation of sodium in adults has been described as 15-22% [16] and, in children, 7% [17].

#### Potassium Intake

The recovery of dietary potassium via urinary excretion is lower than for sodium, with approximately 80-85% of ingested potassium excreted in the urine [18]. Like sodium, some potassium is lost through sweat, but most (~10-15%) is lost via fecal excretion [19,20]. With increased dietary fiber intake, fecal losses of potassium increase [21]. Despite these larger losses, the use of 24-hour urinary potassium excretion is routinely used in population studies to obtain a reliable measure of average potassium intake [7]. Furthermore, assessment of both sodium and potassium via 24-hour urine collection enables the molar ratio of sodium to potassium to be determined.

### Sodium Intake in Children

Internationally, most studies that have utilized a 24-hour urine collection to assess sodium intake were conducted in the 1980s in Europe and in relatively small sample sizes [2]. However, more recent estimates using this robust methodology in larger samples of children come from the United Kingdom (n=340) [22] and Germany (n=364) [17]. In view of the importance of using a reliable measure to monitor population sodium intake, the most recent 2008/09 to 2011/12 UK National Diet and Nutrition Survey included 24-hour urine collections within a sub-sample of children aged 4 years and over [23]. With the exception of one very small study (n=12) completed in 1982 [24], to our knowledge there has been no other attempt to objectively measure sodium intake in Australian schoolchildren using 24-hour urine collection.

The most recent national estimates of sodium intake in Australian children come from 24-hour dietary recall data collected within the 2011-13 Australian Health Survey [25]. In this study, average intakes of sodium from food and beverage sources were high: 2058 mg/d, 2462 mg/d, and 2761 mg/d in 4-8 year olds, 9-13 year olds, and 14-16 year olds, respectively. These intakes exceed the recommended Upper Level (UL) for daily sodium intake [3]. Importantly, as the 24-hour recall measure does not include discretionary salt use [25], it is likely that the total daily sodium intake of Australian schoolchildren is higher. To obtain an accurate assessment of total sodium intake in Australian schoolchildren, the collection of 24-hour urine samples is required.

### Potassium Intake in Children

Very few studies have reported potassium intake in children obtained from 24-hour urine collections [26,27]. Furthermore, to our knowledge, no Australian study has utilized this methodology. Most information relating to potassium intake comes from 24-hour dietary recall data collected during national nutrition surveys [25,28,29]. In Australia, the 2011-13 Australian Health Survey reported average intakes of potassium were 4-8 years: 2138 mg/d; 9-13 years, boys: 2690 mg/d, and girls: 2433 mg/d; 14-18 years, boys: 2830 mg/d, and girls: 2466 mg/d [25]. Overall, average intake falls below the recommended Adequate Intake (AI) for potassium. Of note, due to insufficient data regarding the requirements for potassium in children, the AI was based on population intake estimates from previous national nutrition surveys [3].

### Association Between Sodium and Potassium Intake and Cardiovascular Risk Factors

The dietary electrolytes, sodium and potassium, are both involved in the development of high blood pressure [30]. In adults, excess dietary sodium has a detrimental effect on blood pressure [7] and cardiovascular health [31], whereas higher intake of potassium has been shown to be protective [32]. The interplay between these two electrolytes is important, with the sodium to potassium ratio associated with blood pressure [33,34] and a predictor of cardiovascular disease (CVD) risk [35]. During childhood, sodium and potassium are also important moderators of blood pressure. The evidence for the effects of sodium intake on blood pressure in children and adolescents is strong. Population studies from the United Kingdom [36] and the United States [37,38] have reported a positive association between sodium intake and blood pressure. Furthermore, findings from meta-analyses have shown that reductions in sodium intake lead to modest reductions in blood pressure [4,39].

Findings relating to the effects of potassium alone on blood pressure in children and adolescents are less consistent. For example, some cross-sectional studies have found either a positive association [11,40] or inconsistent findings by gender [41] between potassium intake and blood pressure. Conversely, longitudinal studies have found that a higher intake of potassium is associated with lower systolic blood pressure [6,42]. A recent meta-analysis, which included three intervention trials and one cohort study, found no association between potassium intake and blood pressure levels in children [43]. The World Health Organization’s most recent guideline for potassium intake in children acknowledges the inconsistencies across studies in children. However, given the protective effects of potassium on blood pressure and CVD risk in adults, it is recommended that children’s potassium intake be in line with the recommendation for adults (ie, at least 90 mmol/d or 3510 mg/d) with adjustment for energy requirements [44]. Most studies in children have examined the effects of either sodium or potassium on blood pressure in isolation. One longitudinal study in Dutch children found that although sodium alone was not related to blood pressure, a higher sodium to potassium ratio was predictive of higher systolic blood pressure levels [6].

Raised blood pressure during childhood is associated with target organ damage [45], as well as greater risk of hypertension during adulthood [46]. This emphasizes the need to monitor intake of sodium and potassium during childhood and gain a better understanding of the relationship between these electrolytes and blood pressure levels. Currently, there is no country-specific data assessing the association between an objective measure of sodium and potassium intake with blood pressure levels in Australian schoolchildren.

### Additional Risks of High Sodium Intake During Childhood

Beyond the concerns of high sodium diets raising blood pressure, there is evidence in children to indicate that higher intake of sodium is associated with increased risk of overweight and obesity. There are now data from three large population-based nutrition surveys in children and adolescents (ie, Great Britain [47], Australia [48], and the United States [49]) that support the link between dietary sodium intake and obesity via greater consumption of sugar-sweetened beverages (SSBs). Remarkably similar results have been observed across each country whereby sodium intake was positively associated with SSB consumption, which in turn is an important risk factor for obesity [50]. However, more recent evidence suggests that sodium intake is associated with adiposity, independent of energy intake [51]. To date, no study has utilized a reliable marker of sodium intake (24-hour urinary sodium excretion) to examine the association between sodium intake and measures of adiposity in Australian children. As overweight and obesity follows a trajectory over the life course [52], it is important to understand the association between these risk factors and 24-hour urinary sodium excretion in Australian children.

### Preference for Salt Taste in Children

Taste is an important influence on food choice, particularly in children [53], but there are very few studies worldwide that describe children’s taste preferences for foods with varying levels of sodium. Existing studies with children that included food tasting have used a small number of non-commercial foods, with salt added during preparation [54-57]. Since processed foods contribute a large proportion of dietary sodium [58], and there is variation in preferred saltiness between different food types [59,60], further data across a range of processed foods is needed. This is particularly important as Australia and other countries have categories of food with nominated sodium-reduction targets [61] and consumer acceptance of lower sodium food reformulation is fundamental to achieve sustainable population-wide reductions in sodium. To our knowledge, there is no published research examining the impact of salt on taste preferences in Australian school-age children.

### The Salt and Other Nutrient Intakes in Children (SONIC) Study

The primary aim of the SONIC study was to obtain an accurate measure of total dietary sodium intake using 24-hour urinary excretion in a sample of primary schoolchildren in Victoria, Australia. Secondary aims within this sample of primary schoolchildren were: (1) to objectively measure potassium intake and the sodium to potassium ratio using 24-hour urinary electrolyte excretion, (2) to identify the dietary sources of sodium and potassium, (3) to assess the association between 24-hour urinary sodium and potassium excretion and blood pressure, (4) to assess the association between 24-hour urinary sodium excretion and body mass index (BMI) z-score and waist circumference, and (5) to identify children’s taste preferences and saltiness perception using high and low sodium variants across a range of manufactured foods, which are major dietary sources of salt.

The purpose of this paper is to describe the study design and data collection methods used within the SONIC study. In addition, we report on the recruitment outcomes and data processing procedures for determining completeness of 24-hour urine samples within the study population.

## Methods

### Study Design

The SONIC study was a cross-sectional study conducted within primary schools located in Victoria, Australia from 2009-2013. Ethics approval was obtained by the Deakin University Human Research Ethics Committee (Project No: EC 62-2009). Recruitment and data collection occurred in two phases. The first phase (Phase 1) of the study was conducted within non-government (ie, private) schools during June 2010 to June 2011. Findings from Phase 1 have previously been reported [62]. The second phase (Phase 2) of the study was conducted within government schools during November 2011 to May 2013.

### Rationale for Phase 1 and Phase 2

To enable a representative sample of children across different socioeconomic stratum, this study was initially designed to take place in government schools. However, as this was the first Australian study to propose the collection of 24-hour urine collections within the school sector, there were difficulties in obtaining approval from the governing education authority to conduct the study in government schools. Therefore, to demonstrate the feasibility of collecting 24-hour urines, the study was piloted in a smaller sample of children within the non-government school sector. This was possible as permission to conduct research within non-government schools is granted on a school-by-school basis by either the school board or principal. After the successful completion of Phase 1 within non-government schools, permission to enter government schools was granted by the Victorian Department of Education and Early Childhood Development (2011_001151). Due to the overall difficulties in recruiting schools to participate, we utilized a convenience sampling framework.

### Participants

Participants were children attending non-government and government primary schools in Victoria, Australia. Primary school-aged children were targeted, as opposed to adolescents in high school, as findings from the 2007 Children’s Nutrition and Physical Activity Survey (CNPAS) revealed younger children had particularly high sodium intake compared to dietary recommendations [3,63]. To maximize recruitment, participation was open to all children attending the primary school. However, in some instances, the school principal thought it was inappropriate for very young children (eg, prep classes or grade 1) to participate in the study. Therefore, to respect the requests of individual schools, the grades invited to participate varied across schools. To help engage families to participate in the study, parents of participating children had the option of completing a 24-hour urine collection. Children were excluded from the food tasting session if they had food-related anaphylaxis. Those children with food specific allergies (eg, gluten intolerant) were not permitted to taste relevant food products (eg, bread).

### Recruitment

#### Phase 1

**Figure 1:**
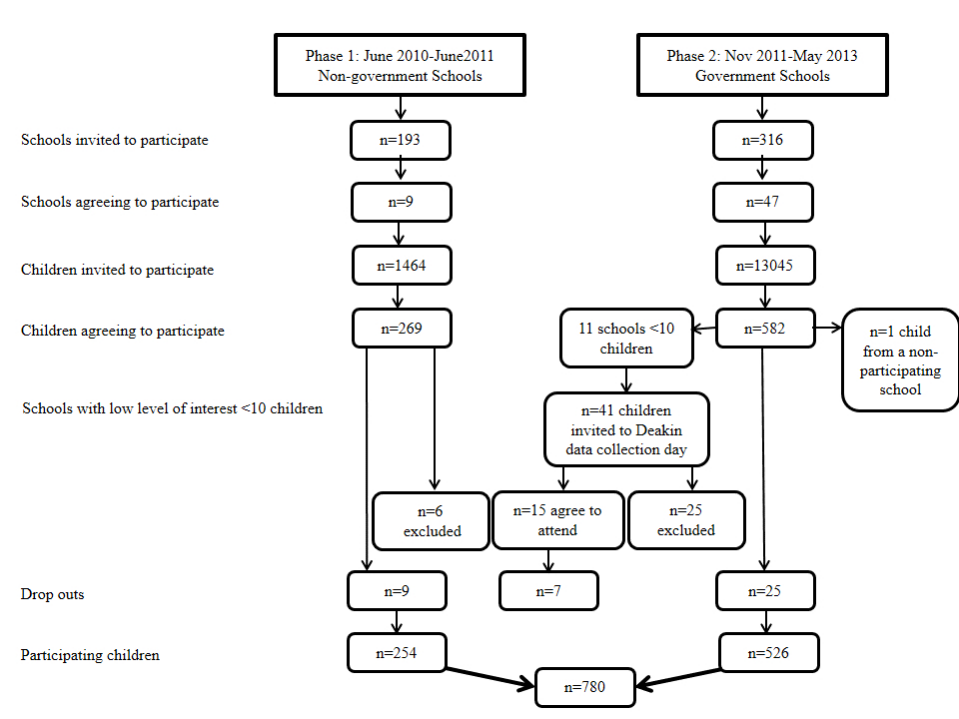
Flow and response rates of participants in the SONIC Study.

Non-government privately funded schools located in Victoria were targeted for recruitment. A Web-based school locater search engine was used to identify all those non-government Victorian schools with enrollments of primary school children (n=193) [64]. A convenience sample of schools was selected from the list, and principals were contacted via an official letter or email of invitation and a phone call, inviting school participation in the study. In total, 104 schools were invited, of which 9 agreed to participate in the study (response rate=8.7%) (Figure 1). A short presentation, outlining the purpose of the study, was presented to students at the school assembly, after which students were provided with study information packs containing written materials, addressed to parents, inviting them to take part in the study. Reminder notices, outlining the purpose of the study and the procedure to participate, were also included in school newsletters. Of the potential 1464 students, 269 agreed to participate (response rate=18.4%); nine of these children later dropped out of the study. Reasons for attrition included no longer interested in participating (n=3), being absent on the day of data collection (n=5), or no longer attending the school (n=1). Data from six participating children were excluded from analysis as they were 13-14 years of age (ie, in grade 8 of high school) and the target group for this study was primary school. These children were invited to participate as they were enrolled at a unique school that included children from grade preparatory-10. Written consent was obtained from the child, as well as the child’s parent/caretaker. To thank children for their participation time, an AU $20 book voucher was provided.

#### Phase 2

Victorian government schools with enrollments for primary schoolchildren were identified using the Department of Education and Early Childhood Development online school locator (n=649) [65]. At the commencement of recruitment in September 2011, Victorian government schools were divided into four regions (Northern, Eastern, Southern, and Western Metropolitan regions); since the time of recruitment, these regions have changed slightly. A convenience sample of schools was selected, starting within the Eastern Metropolitan region, due to the proximity to Deakin University Burwood Campus. Once school recruitment was exhausted within the Eastern region, the Southern Metropolitan region was sampled, followed by the Northern Metropolitan region. No schools in the Western Metropolitan region were contacted as the study had reached its funding capacity. As per Phase 1, principals were contacted via an official letter or email of invitation and a phone call, inviting school participation in the study. In total, 316 were invited, of which 47 agreed to participate (response rate=14.9%) (Figure 1).

The recruitment strategy for children differed between participating government schools, due to constraints placed on the amount of access the project officer could have within the schools. For example, it was not always possible to do the short presentation outlining the study at all schools. Furthermore, study information packs were not distributed to all children at the school. Instead, a “recruitment starter pack”, which contained study information packs, was delivered by the project officer to the school principal, who then organized distribution of the packs to school children. Across all schools, the study was advertised in the school newsletter and parents could then contact the project officer via email or phone expressing their interest in the study and request an information pack. Of the potential 13,045 students enrolled at schools and invited to participate, 582 agreed to participate (response rate=4.5%). To avoid high costs associated with travelling to schools with only a few participants, data collection procedures occurred only at schools with ≥10 participants. In schools (n=11) with <10 participants, children were advised that to participate they would have to travel to Deakin University in Burwood, Melbourne to complete data collection procedures on a Saturday. Of the 41 children that this applied to, 15 agreed to visit Deakin University; however, 7 of these did not attend (ie, dropouts) on the scheduled day of data collection. The remaining 25 were excluded from the study as they were not willing to come to Deakin University. A further 25 children dropped out of the study; reasons for attrition included no longer interested in participating (n=7) or being absent on the day of data collection (n=18). Finally, one child from a school that refused to participate attended the Deakin University data collection day. This child was aware of the study as their mother worked at Deakin University. Written consent was obtained from the child, as well as the child’s parent/caretaker. Due to guidelines for inducements for study participation in government schools, either a book voucher to the value of AU $20 per participant or $100 per school was issued to the school library to thank the children for their participation.

### Data Collection

With the exception of the one day of data collection completed at Deakin University, testing procedures were completed at schools by a team of research staff. In Phase 1 of the study, data was collected at participating schools across two time blocks, June-December 2010 and May-June 2011. In Phase 2 of the study, data was collected as schools were recruited between November 2011 and May 2013.

### Testing Procedures

An overview of data procedures completed is shown in Table 1.

**Table 1:** Overview of data collected.

Item		Description
Health and medical questionnaire		Child’s date of birth, gender, birth weight, health conditions, medications, dietary supplements, discretionary salt use habits, parent’s highest level of education attained
Anthropometry		Height, weight, body mass index, waist and hip circumference
Blood pressure		Systolic and diastolic blood pressure
24-hour urine collection		Urinary sodium, potassium, creatinine, total volume
24-hour dietary recall		Food and beverage intake, table salt use
Taste testing		Preference and discrimination with high vs lower salt food item
**Parental component (optional)**		
	Demographics	Date of birth, gender
	24-hour urine collection	Urinary sodium, potassium, creatinine, total volume

### Demographic Characteristics

The parent of each child completed a health information questionnaire (see Multimedia Appendix 1). The questionnaire collected information on the child’s age, gender, birth weight, existing medical conditions, and use of medications or dietary supplements. In Phase 2 of the study, the questionnaire was modified to include an additional question relating to the parent’s highest level of education attained and information on this was retrospectively collected from parents with children participating in Phase 1.

### Discretionary Salt Use

Both parents and children were asked to report on the frequency of discretionary salt use. For parents, three questions were included on the health information questionnaire, two of which applied to the parent’s use of salt, “Q1: Do you add salt during cooking?” (used previously in 2007 CNPAS [66]) and “Q2: Do you place a salt shaker on your table at meal times?”, and one question that related to the child’s own use of salt at meal times, “Q3: Does your child add salt to their meal at the table or sandwich preparation?” For each of these questions, parents could respond “yes usually”, “yes sometimes”, “no”, or “don’t know”. At school on the day of data collection, the study child was also questioned about their own use of table salt. Children were asked, “Does the person who prepares your meal add salt when cooking?” and “Do you add salt to your meal at the table?” (used previously in 2007 CNPAS [66]), to which they could respond “yes, usually”, “yes, sometimes”, “no”, or “Don’t know”.

### Anthropometry

Height, body weight, waist, and hip circumference were measured according to the protocols of the International Society for Advancement of Kinanthropometry [67]. For all measures, children were wearing light clothing with shoes removed. Height was measured to the nearest 0.1 cm using a calibrated portable stadiometer SECA (mod 220) (Hamburg, Germany). Weight was measured to the nearest 0.1 kg using a calibrated FS-127-BRW portable electronic scale (Bradman, MA, USA). A minimum of two measurements were taken for height and weight. If the two measurements were not within 5 mm for height or 0.1 kg for weight, a third measurement was taken. The mean value was used as the final score if two measurements were taken. The median value was used as the final measure if three measurements were taken. BMI was calculated as body weight (kg) divided by the square of body height (m^2^). Age and gender adjusted BMI *z*-scores will be calculated using the LMS method [68] with the 2000 US Centers for Disease Control and Prevention (CDC) Growth Charts acting as the reference population [69]. Participants will be grouped into weight categories (very underweight, underweight, healthy weight, overweight, obese) using the age and gender-specific International Obesity Task Force BMI reference cut-offs for children [70,71].

Waist and hip circumference was measured to the nearest 0.1 cm using a Lufkin Executive Thinline W606PM pocket tape (Sparks, MD, USA). Waist circumference was taken at the end of a normal expiration at the narrowest point between the lower costal border and the top of the iliac crest. Hip circumference was measured at the level of the maximum protrusion of the gluteal muscles. A minimum of two measurements were taken for waist and hip circumference. If the two measurements were not within 10 mm of the first, a third measurement was taken. The mean value was used as the final score if two measurements were taken. The median value was used as the final measure if three measurements were taken.

### Blood Pressure

Blood pressure was measured using an automatic vital signs monitor (OMRON HEM-907) machine according to the US National Heart Lung and Blood Institute Guidelines [72]. Measurements were completed in the sitting position after 10 minutes of rest with the child’s arm positioned at the level of the heart on a rested table. Children were instructed to refrain from talking or coughing during the measurements. To determine the appropriate cuff size, the child’s upper arm (midpoint between the acromion and olecranon) circumference was measured and from this one of three cuff sizes (small 17-22 cm, medium 22-32 cm, large 32-42 cm) was selected for use. The cuff was positioned on the child’s arm by aligning the marked cuff artery indicator with the brachial artery. Once wrapped firmly on the arm, the cuff size was checked by ensuring the marked index line fit within the specified range line on the cuff. If the cuff was noted as being too small or too large, the size up or down was fitted. Three blood pressure readings were taken on the right arm at 1-minute intervals. The average of the last two measurements will be used for analysis.

### Dietary Recall

A three-pass 24-hour dietary recall was used to determine all food and beverages consumed from midnight to midnight on the day prior to the interview. To be consistent with the protocols used in the 2007 CNPAS survey, we utilized the three-pass method. This method includes the following stages: (1) provide a quick list of all foods and beverages, (2) a series of probe questions relevant to each quick list item to gather more detailed information including quantifying the amount consumed, the time and place of consumption, any additions to the food item, brand name, and meal occasion (ie, breakfast, morning tea, lunch, afternoon tea, dinner or supper), and (3) finally, a recall review to validate information and make any necessary adjustments. Portion sizes were estimated using a validated food model booklet and standard household measures. All items were hand-recorded on a food intake form by research staff with a nutrition background and who had received training from an Accredited Practicing Dietitian. It is recognized that children aged 8 years and above have the cognitive ability to recall their own food and beverage intake, whereas in younger children there is a need for a proxy (eg, parent) to help recall intake [73]. In this study, 24-hour recalls were completed on the day of testing at an allocated space within the school. Up to six children were present within each testing session, with children rotating across data collection stations. To avoid young children feeling excluded from certain collection procedures, all children completed the 24-hour dietary recall. However, in analyses related to dietary assessment only recalls in children aged 8 years and above will be utilized. The 24-hour food and beverage intakes will be converted into nutrient intakes, using FoodWorks Version 8, which is linked to the Australian nutrient composition database AUSNUT 2011-13 [74]. Daily sodium (g) intake will be converted to the salt equivalent (g) using the conversion factor 2.543 [18].

The Goldberg cut-off method [75], adjusted for use within the pediatric population [76], will be used to identify participants with implausible energy intakes. Child-specific physical activity levels (PAL) [77] will be used to calculate age and gender-specific Goldberg cut-offs. Schofield equations, specifying age, gender, body weight, and height [78], will be used to calculate each participant’s estimated basal metabolic rate (estBMR). The ratio of each participant’s reported energy intake to estBMR (EI:estBMR) will be compared to the appropriate calculated age and gender-specific Goldberg cut-off value. A participant with an EI:estBMR below the cut-off value will be classified as a low energy reporter.

### Taste Testing Procedures

**Figure 2:**
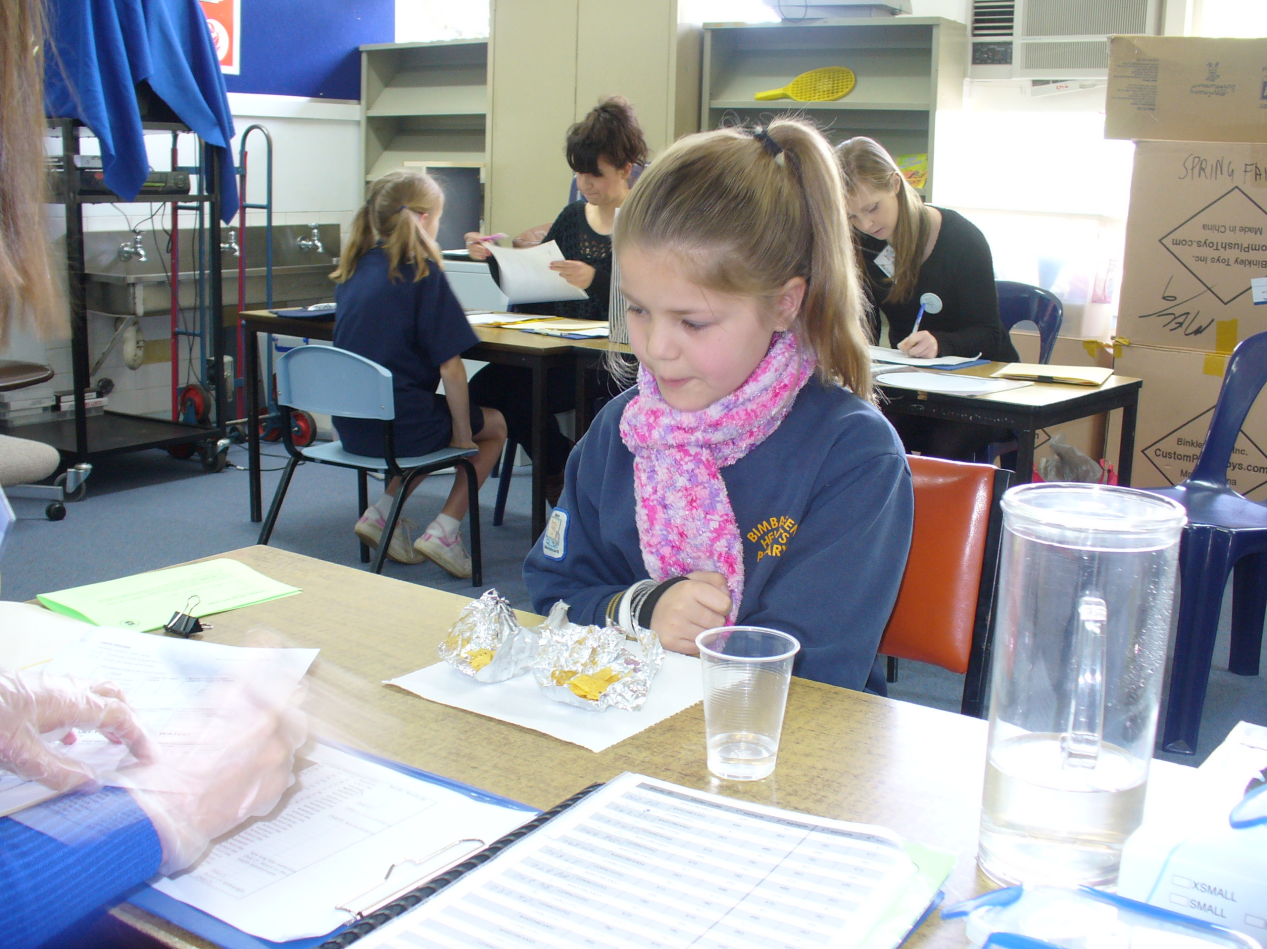
SONIC study participant completing data collection procedures.

Tastings were conducted in one-to-one sessions using pre-packed, commercially available high and low salt variants of processed foods that have been identified as major sources of dietary sodium in Australian children (Table 2) [79]. A variety of different core food items such as bread, cereals, and cheese were evaluated. Within core food types tested, the difference in sodium content of the two samples varied between 27% and 84%. For example, one brand of bread with a sodium level of 202mg/100g was compared to another with 511mg/100g (60% difference). There were also differences in the percentage sodium variation within food categories, for example, processed meats varied between 25% and 40%, while for snack foods, potato crisps varied by 94% and tomato sauce by 27%. Differences in sodium content were calculated based on sodium levels provided on package nutrition information panels. To assess taste preference, children were asked to taste high and low salt samples of a food (Figure 2), and asked which they preferred using a forced choice methodology previously shown to be appropriate for use with children [80]. After this, they were asked which of the samples they thought tasted saltier. Participants were then asked to drink some water, and the same process was repeated with a second food type. The two food types tested, with two samples for each, were presented in unmarked wrapping in randomized order. Pairs of low and higher sodium foods were matched as closely as possible for appearance and nutritional composition other than sodium level.

**Table 2:** Foods tested in sensory component.

Bread and cereal	Sandwich fillings	Snack foods
Bread: sourdough white; sourdough wholemeal^b^	Cheese, sliced cheddar^a^	Crisps, potato^a^
Wraps, multigrain^a^	Ham: sliced^a^	Corn chips, plain^a^
Cornflakes^b^	Chicken, sliced roast^a^	Rice crackers, plain^a^
Wheat cereal biscuits^a^		Baked beans^a^
		Tomato sauce^a^

^a^One-paired comparison, using low/high salt food variants.

^b^Two-paired comparisons, using low/high salt and medium/high salt food variants.

### 24-Hour Urine Collection

#### Collection Protocol

Participants could commence the 24-hour urine collection either at school or at home over the weekend. Written instructions, tailored for either a school or weekend day collection, were provided for parents, and simplified pictorial instructions were provided for children. All children were instructed to commence the collection by emptying their bladder, discarding this urine, and noting this as the 24-hour collection start time. During the 24 hours following this, all urine voided was collected. Children finished the collection with a final void at the corresponding 24-hour finish time. Urine was collected in 2.5 L wide-mouth, rimmed polypropylene bottles. To assist with urine collection, an additional 500 mL plastic handled jug was provided. Children were asked to report any missed collections or spillages on a urine collection slip, which was returned with the 24-hour urine sample.

School day collections commenced during school hours and at the end of the school day children collected their materials to continue the collection at home. On the following school day, children returned to research staff and finished the collection by providing a final void. Children completing the collection on a weekend day were able to commence the urine collection at any suitable 24-hour period over the weekend, parents recorded start and finish times on the 24-hour urine bottle and a urine collection slip, children were instructed to return completed samples to research staff at school on the following Monday morning.

#### Urinalyses

Urinary sodium and potassium concentration was assessed using indirect ion selective electrodes and urinary creatinine concentration was assessed using the Jaffe reaction [81] on the Siemens Advia 2400 analyzer (Dorevitch Pathology, Melbourne, Vic, National Association of Testing Authorities and Royal College of Pathologists of Australia accredited pathology laboratory). Per participant 2 x 10 mL aliquots were taken for storage and transferred to −80 ^°^C conditions. The molecular weights of sodium (23 g/mol), sodium chloride (58.5 g/mol), and potassium (39.1 g/mol) will be used to convert laboratory of mmol to mg [18].

### Criteria for Assessing Completeness of 24-Hour Urine Samples

**Figure 3:**
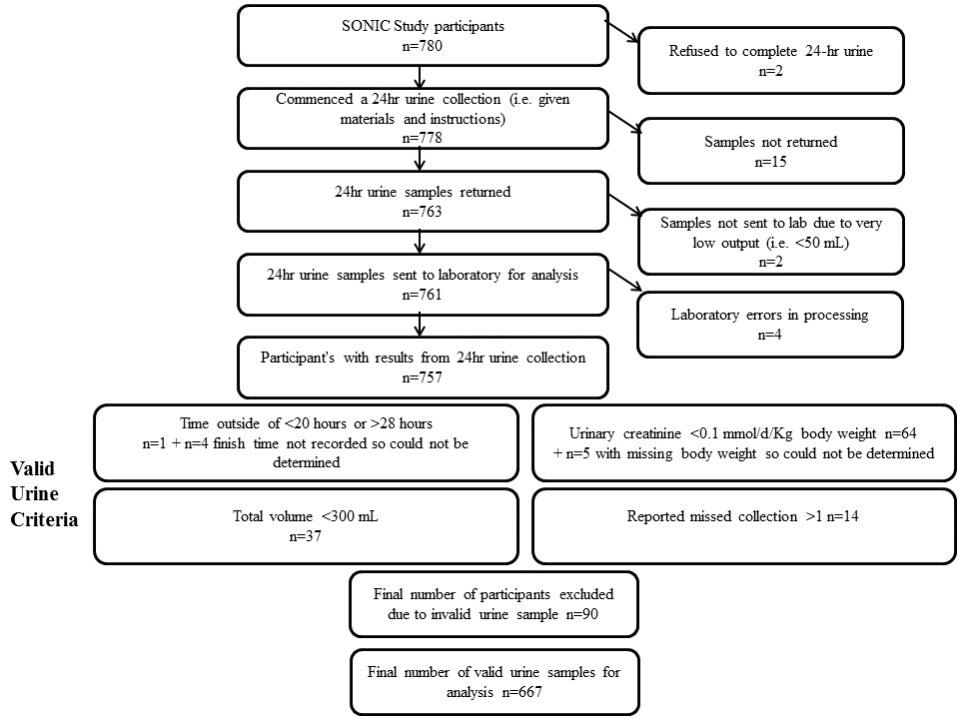
Flow chart of assessment of valid 24-hour urine collections for analysis.

If the duration of the collection was not exactly 24 hours (but within 20-28 hours), urinary sodium, potassium, creatinine, and total volume were normalized to a 24-hour period. Following this, urine collections will be considered incomplete and excluded if (1) the timing of the collection was <20 hours or >28 hours, (2) total volume was <300 mL, (3) the participant reported missing more than one collection, or (4) urinary creatinine excretion was less than 0.1 mmol/Kg body weight/day [22,41,82]. A total of 15 participants did not return their urine collection slip, so it was not possible to confirm if they had missed collections or spillages. In these children, the completeness of the 24-hour urine collection was assessed based on the other three criteria. Of the 780 participants, 757 (97.1%) had available results from the 24-hour urine collection (Figure 3). Based on the completeness criteria, 90 participants (11.5%) were classified as providing invalid 24-hour urine samples and will be excluded from analyses. Note the number of invalid urines based on the four criteria (n=125) is greater than the final number of excluded urines (n=90) as some participants’ 24-hour urine collection met more than one of the criteria for invalid urine (Figure 3).

### Sample Size

To estimate mean sodium intake within a 95% confidence interval of +/− 4 mmol/d and assuming a standard deviation of 37 mmol/d (unpublished 24-hour urine pilot data from UK children) [83], a sample size of 329 children was required. We anticipated recruiting 10 children from each school (ie, cluster size n=10) and assumed an intra-cluster correlation (ICC) of .01, giving a design effect (DEFF = 1 - (n-1) ICC) of 1.09. Therefore the sample size after adjusting for clustering was 359 children. Allowing for a 10% loss due to dropout and incomplete urine samples, the target sample size was therefore 395. Given gender differences in sodium intake, we aimed to recruit 395 boys and 395 girls (ie, total n=790).

### Data Analysis

Descriptive statistics will be used to describe sodium and potassium intakes. Dietary sources of sodium and potassium will be determined at the individual level using the mean ratio method [84]. Liner and logistic regression models will be used to assess the association between electrolyte intake and health outcomes (blood pressure and BMI *z*-score and waist circumference) and to assess differences in taste preference and saltiness discrimination between food types, and correlations between taste preference, sodium intake, and covariates. To account for clustering of students within schools, clustered robust standard errors will be used. Analyses will be adjusted for covariates (age, gender, socioeconomic status).

## Results

Data collection was completed in May 2013. A total of 780 children across 43 schools participated. Data analysis is currently underway and results are expected at the end of 2015.

## Discussion

### Strengths and Limitations

This study will provide the first objective measure of sodium and potassium intakes in Australian schoolchildren. The major strength of this study is the use of 24-hour urine collections to assess total daily sodium intake in a large sample of schoolchildren. Furthermore, a well-devised 24-hour urine collection protocol, tailored specifically to children was used. Limitations of the study include the convenience sampling framework and the low response rate, which limits the generalizability of the study findings. In addition, dietary recall data is only available on those children aged 8 years and above and only 1 day of recall data was collected.

### Conclusion

Given the cardiovascular implications of consuming too much sodium and too little potassium, monitoring of these nutrients during childhood is an important public health initiative. The methods described within provide a platform for other research groups and public health organizations to conduct 24-hour urinary sodium and potassium measurements in wide-ranging children and potentially adolescent populations, thus adding to the evidence base exploring the intake of these important electrolytes, their implications of health in the early stages of life, and the potential impact on food choice.

## Multimedia Appendix 1

Health information questionnaire.

## Abbreviations

AI: adequate intake
BMI: body mass index
CNPAS: Children’s Nutrition and Physical Activity Survey
CVD: cardiovascular disease
EI: estBMR: energy intake:estimated basal metabolic rate
estBMR: estimated basal metabolic rate
ICC: intra-cluster correlation
SONIC: Salt and Other Nutrient Intakes in Children study
SSB: sugar-sweetened beverage
