# Supplementary material for: Cross-Sectional Study of 24-Hour Urinary Electrolyte Excretion and Associated Health Outcomes in a Convenience Sample of Australian Primary Schoolchildren: The Salt and Other Nutrients in Children (SONIC) Study Protocol
Source: JMIR Res Protoc. 2015 Jan 15;4(1):e7. doi: 10.2196/resprot.3994 (PMC4319086; doi:10.2196/resprot.3994)
Supplement: Supplementary file 1 [file resprot_v4i1e7_app1.pdf]

Child's Name: _________________________ *(this information will be removed once returned)*

SONIC Study

Subject ID: _____________

School ID: _____________

Salt & Other Nutrient Intakes in Children

In this questionnaire, you will be asked questions about your child's general background, health status and salt intake. Please allow approximately 10 minutes to complete this questionnaire. All responses will remain confidential. Please return the completed questionnaire with consent form in the envelope provided to your child's class teacher.

SECTION 1 - PERSONAL INFORMATION

Today's Date: ___ /___ /____ (dd/mm/yy)

Q1.1 What is **your** highest level of schooling? (Tick one box)

- Never attended school ☐1
- Primary school ☐2
- Some high school ☐3
- Completed high school ☐4
- Technical/Trade certificate ☐5
- University or Tertiary level ☐6

Q1.2 Date of birth of child: _____ / _____ / _____(dd/mm/yy)

Q1.3 Gender of child (please tick) Female ☐1 Male ☐2

Q1.4 Looking at your baby's Child Health Record (yellow book), please go to the Birth, Vitamin K and Hepatitis B, Newborn Examination section, and record your child's weight at **birth** _________grams

PTO ⇨

All questionnaires are confidential

SECTION 2 – HEALTH INFORMATION

Q2.1 Does your child have any health or medical conditions? (Tick box):

☐1 No

☐ Yes - If yes, please explain below

____

____

____

____

Q2.2 Is your child currently taking any medications? (Tick box):

☐1 No

☐2 Yes - If yes, please specify the **NAME** of the medication, the **DOSE**, the **DURATION OF USE** and the **REASON** for using the medication

**Name of medication**	***Dose***	***Frequency (per day)***	***Duration***	**Reason for taking medication**
Example<br>*Femiron*	*Example*<br>*100mg*	*Example*<br>*once a day*	*How long has child been taking medication?*<br>*Example: 6 weeks*	*Example*<br>*anaemia*

PTO

All questionnaires are confidential

SECTION 2 – HEALTH INFORMATION (continued)

Q2.2 Is your child currently taking any dietary supplements? (Tick box):

☐1 No

☐2 Yes - If yes, please specify the **NAME** of the dietary supplement, the **DOSE**, the **DURATION OF USE** and the **REASON** for using the medication

**Name of dietary supplement**	***Dose***	***Frequency (per day)***	***Duration***	**Reason for taking supplement**
Example<br>*Omega 3 Fish Oil – Select Lifestyle*	*Example*<br>*1000mg*	*Example*<br>*once a day*	*How long has child been taking supplement?*<br>*Example: 6 months*	*Example*<br>*General well-being*

PTO

SECTION 3 – YOUR CHILD'S SALT INTAKE

(This information will be used to supplement the dietary data provided by your child)

Q3.1 Do you add salt during cooking?

Yes, usually ☐ 1

Yes, sometimes ☐ 2

No ☐ 3

Don't know ☐ 4

Q3.2 Do you place a salt shaker on your table at meal times?

Yes, usually ☐ 1

Yes, sometimes ☐ 2

No ☐ 3

Don't know ☐ 4

Q3.3 Does your child add salt to their meal at the table or sandwich preparation?

Yes, usually ☐ 1

Yes, sometimes ☐ 2

No ☐ 3

Don't know ☐ 4

Thank you for completing the questionnaire. Please return the completed questionnaire with the consent form to your child's class teacher
